# Paradoxical Features Empower Biogenic Silver Nanoparticles

**DOI:** 10.3390/molecules30214152

**Published:** 2025-10-22

**Authors:** Jackeline Pereira, Otto Proaño, Andrea Albán, Marjorie Zambonino, Lynda Mouheb, Morgane Desmau, Ashiqur Rahman, Spiros N. Agathos, Si Amar Dahoumane

**Affiliations:** 1 School of Biological Sciences and Engineering, Yachay Tech University, Hacienda San José s/n, San Miguel de Urcuquí 100119, Ecuador; pampereirac2804@gmail.com (J.P.); otto.proano@hotmail.com (O.P.); andreitaalban@hotmail.es (A.A.); marjorie.zambonino@gmail.com (M.Z.); 2 Laboratoire de Recherche de Chimie Appliquée et de Génie Chimique, Hasnaoua I, Université Mouloud Mammeri B.P.17 RP, Tizi-Ouzou 15000, Algeria; lynda.mouheb@ummto.dz; 3 Department of Chemistry and Biochemistry, Université de Moncton, 18, Ave Antonine Maillet, Moncton, NB E1A 3E9, Canada; morgane.desmau@umoncton.ca; 4 Center for Midstream Management and Science, Lamar University, Beaumont, TX 77710, USA; ashiqur.1912@gmail.com; 5 Earth and Life Institute, Catholic University of Louvain, B-1348 Louvain-la-Neuve, Belgium; spiros.agathos@uclouvain.be; 6 Qingdao Innovation & Development Base, Harbin Engineering University, Qingdao 266000, China

**Keywords:** silver nanoparticles, biosynthesis, sustainability, biocompatibility, coating biomolecules, biological properties, biomedical applications, bioremediation

## Abstract

Silver nanoparticles (AgNPs) have drawn great attention, owing to their unique physico-chemical and biological properties and various applications, particularly in the biomedical field. In addition to conventional chemical and physical methods, materials scientists have been exploring the capabilities endowed by several bioresources, such as plants, bacteria, fungi and algae, in the cost-effective and eco-friendly production of AgNPs. This review article provides a comprehensive overview of the current state of research on the bioapplications of biogenic AgNPs (*bio*-AgNPs). The various bioresources used and methodologies followed to synthesize *bio*-AgNPs are briefly examined, along with some aspects of the underlying mechanisms. Then, the review surveys the toxicity of AgNPs, in general, and presents the unique biological properties of *bio*-AgNPs. Furthermore, the review details numerous applications of *bio*-AgNPs with paramount importance to human health, such as the control of infectious disease vectors, cancer therapy, antibiofilm activity and environmental remediation. Importantly, the review highlights the paradoxical effect of these nano-objects since they specifically seem to exert their action solely on targeted cells and (micro)organisms. By featuring the unique advantages of biogenic methods and their challenges, this article aims at serving as a valuable resource to attract research on *bio*-AgNPs and elicit further developments towards the scalable and sustainable production of AgNPs for large scale industrial and clinical use.

## 1. Introduction

Nanoparticles (NPs) are structures that have at least one dimension at the nanoscale, i.e., spanning from 1 to 100 nm [1,2,3,4]. They are 0-D (zero-dimensional) if all the dimensions are at the nanoscale [2,3,4]. Nanospheres, nanocubes and quantum dots are some examples of 0-D NPs. One-dimensional NPs have two dimensions at the nanoscale while the last one exceeds 100 nm [2,3,4]. This is the case, for instance, of nanorods and nanotubes. Two-dimensional NPs, such as nanoplates, nanofilms, nanolayers and nanocoatings, exhibit only one dimension at the nanoscale [2,3,4]. Lastly, 3-D NPs constitute a special category since they have no dimension at the nanoscale. However, their texture reveals the presence of features, such as spines or holes, at the nanoscale, which ensures them the classification as a nanomaterial [2,4]. Nano-urchins and nano-wells are examples of 3-D NPs.

Nanomaterials can be biological, organic, inorganic and hybrid [5,6]. Inorganic NPs have attracted significant attention across diverse fields owing to their unique properties that have given rise to an ever-growing body of research enabling their application in various fields and triggering vigorous advancements in multiple domains [7,8,9,10,11,12,13]. In the metallic category, silver NPs (AgNPs) have been extensively studied, owing to their remarkably unusual physical, chemical, and biological properties that are directly influenced by their size, shape, and surface chemistry. AgNPs are exploited for multiple applications, including the biomedical field, agriculture, the food industry and the environment [14,15,16,17]. In the biomedical field, AgNPs exhibit outstanding potential to control the growth of or eliminate various disease-causing organisms, such as pathogenic bacteria and a broad spectrum of viruses [15,18,19,20,21]. Additionally, AgNPs have witnessed exciting developments in wound healing, burn injuries, coatings for implants, drug delivery, biosensing and bioimaging in addition to cancer therapy [6,22,23,24,25].

In drug delivery systems, AgNPs offer unique advantages, such as adjustable size, large surface area, and tunable surface chemistry, allowing the efficient attachment of both drug molecules and recognition moieties, and subsequently the targeted and controlled delivery, and monitored release, of therapeutic agents [6,26,27,28]. AgNPs have also demonstrated promise in bioimaging due to their strong and tunable plasmonic properties, enabling enhanced contrast in various imaging modalities, such as optical imaging, computed tomography (CT), and photoacoustic imaging [29,30,31]. Furthermore, AgNPs have been extensively explored in biosensing applications due to their high sensitivity, selectivity, and stability, facilitating the detection of pathogens, biomarkers and other bioanalytes in very tiny concentrations, owing to their unique optical properties and outstanding surface enhanced Raman spectroscopy effect [29,32,33,34,35]. In the environment, AgNPs are used, for instance, in wastewater treatment, sensing of heavy metals, and photodegradation of dyes and colorants [36,37,38,39].

The present review surveys first the various methodologies developed to produce biosynthetically silver NPs (*bio*-AgNPs), summarizes some of the key mechanistic aspects that govern their toxicity, and extensively discusses their bioapplications in the biomedical and environmental fields. Importantly, it highlights, using specific examples, their paradoxical effects since these nano-objects may exhibit the desired activity against the target microorganisms/cell lines while they remain devoid of any unwanted side-effects towards the untargeted counterparts. This review also discusses the use of *bio*-AgNPs in the degradation of environmental pollutants like organic dyes. Finally, the findings are summarized, and some exciting emerging perspectives are provided.

## 2. Biosynthesis of AgNPs

To synthesize AgNPs, various chemical and physical methods are followed, such as sonochemistry, photochemistry, microwave chemistry, laser ablation and ball milling to name a few [30,40]. Some of these chemical routes may rely on costly and/or hazardous chemicals whereas their physical counterparts may require the use of sophisticated equipment in addition to issues encountered upon scaling up the NP production [41]. Chemically produced AgNPs offer many advantages, such as cost-effectiveness and scalability, shape and size control, adequate surface chemistry, and versatility to potentially find various applications in several fields [20,42,43,44]. However, some limitations restrict their integration into the market for many reasons, such as the use of toxic reagents and/or the generation of hazardous byproducts during the fabrication and/or the application stages, rendering them highly toxic or, at least, not sufficiently biocompatible [40,45].

**Figure 1:**
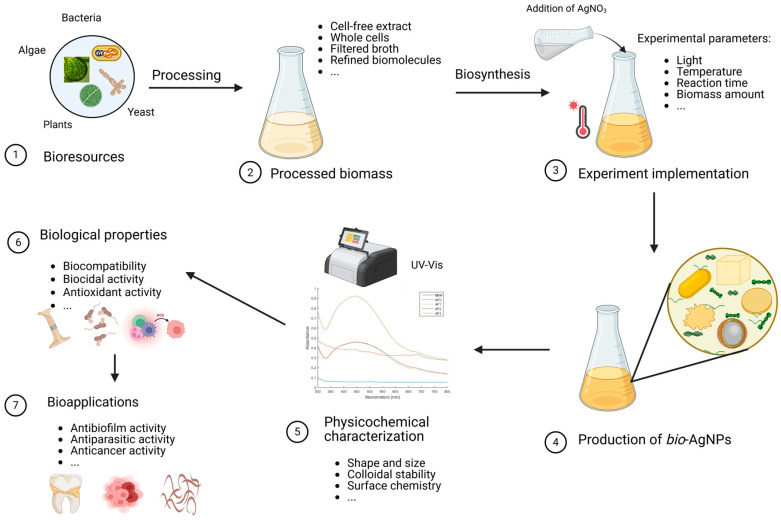
Biosynthesis of silver nanoparticles. The first step consists of preparing the biomass. For instance, the relevant parts of the plants are directly harvested while the microorganisms (bacteria, fungi, microalgae) are cultured ①. Then, the biomass is processed according to the envisaged pathway ②. Subsequently, this bioresource, that can be made of the crude broth, refined biomolecules, disrupted cells, cell-free supernatant, or pristine cultures, is challenged by the precursor (aqueous solution of cationic silver in this case) under a set of selected experimental parameters (pH, temperature, reaction time, etc.) ③. This gives rise to *bio*-AgNPs, ④ whose features (size, shape, stability, etc.) are studied using a variety of characterization techniques ⑤. Then, the biological properties of these NPs are explored ⑥ from which some interesting bio-applications may emerge ⑦.

To overcome these challenges, greener, sustainable and scalable methodologies have been devised to synthesize a very large variety of nanomaterials, among which AgNPs hold a special position [46,47,48]. These well-established yet fast-growing routes rely on living microorganisms, such as algae, bacteria, fungi, yeast, their extracts, plants’ extracts, and combinations of biomolecules to promote the fabrication of AgNPs (Figure 1). These processes fit within the bottom–up approach and, most often, meet several principles of Green Chemistry since they are easy to implement and are, usually, carried out in aqueous media at atmospheric pressure and room temperature or with mild heating, do not rely on any added chemicals except the precursors, exploit renewable biomolecules, and do not require sophisticated equipment [49,50,51]. When the organisms are directly used without any further processing, these routes are coined intracellular since the synthesis occurs inside the cells, as reported by Klaus et al. who carried out the synthesis of AgNPs in the shape of spheres, triangles, and hexagons, using the silver-resistant bacterium *Pseudomonas stutzeri* grown on AgNO_3_-containing agar substrate [52]. A similar procedure was implemented by other groups [53]. On the other hand, the process is termed extracellular when the synthesis occurs outside the cells thanks to the different metabolites released by the cells into the supernatant [54,55,56]. However, the epithet ‘extracellular’ is somehow flagrantly used in the literature to qualify the cell-free synthesis of NPs using processed microorganisms as extracts in reaction media devoid of any cells [57].

Several reviews have been fully or partially dedicated to the biosynthesis of AgNPs [58,59,60,61,62]. As one of the most popular methodologies, the cell-free pathway is highly advantageous since it is simple to implement and might be time- and cost-effective, especially when it utilizes extracts of plants, such as those derived from leaves, stems, or fruits, extracts of fungi, bacteria or algae and enables easy control over the environment in which the NPs are produced [58,63,64,65]. For instance, the polysaccharide-rich, cell-free supernatant of the green microalga *Chlamydomonas reinhardtii*, is used to produce *bio*AgNPs starting from aqueous solutions of cationic silver via a light-driven process [55]. However, aqueous suspensions of washed cells of the same microalga that are devoid of polysaccharides keep their bioreducing capabilities, although the obtained AgNPs lack any colloidal stability and display various shapes and forms [55]. Thus, this study highlights the double role that polysaccharides may fulfill during the biosynthesis of AgNPs: light-activated bioreducing agents and stabilizing moieties that protect the NP shape and size from any alteration. These findings regarding the stabilizing role played by the polysaccharides corroborate another study reporting the sedimentation of gold NPs made by *Euglena gracilis*, a microalga that does not produce polysaccharides [66].

**Figure 2:**
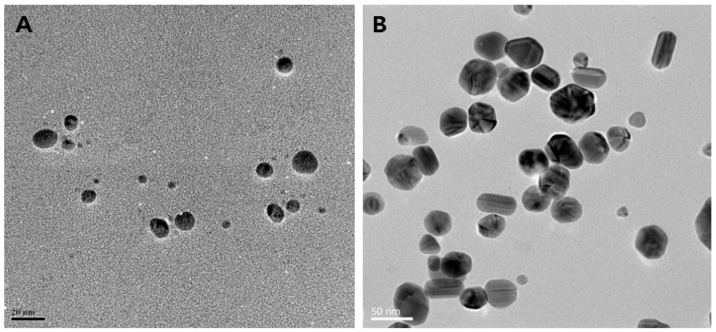

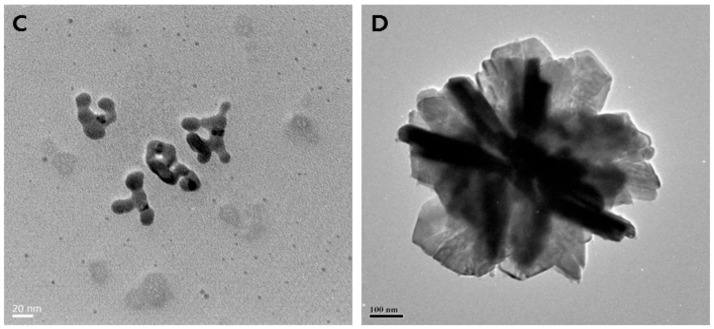
Biosynthesis of different shapes of AgNPs using the supernatant of cultures of various *Bacillus* species. (**A**) Spherical (scale bar: 20 nm); (**B**) mixed populations (octagonal, rod, hexagonal, and icosahedral) (scale bar: 50 nm); (**C**) highly branched (scale bar: 20 nm); (**D**) flower-like in shape (scale bar: 100 nm). Reproduced from Ref. [76] with permission from MDPI under Creative Commons CC BY 4.0.

Various experimental conditions have been screened for the cell-free synthesis of AgNPs, such as the pH, temperature, reaction time, and concentration of silver precursor and biomass content, resulting in desired morphologies and forming spherical, triangular, or hexagonal NPs [67,68,69,70,71,72,73]. For instance, AgNPs obtained using the cell-free supernatant of different strains of *Bacillus* sp. exhibit different shapes, as displayed in Figure 2. The underlying mechanistic aspects involve first the reduction of silver ions into their metallic counterparts by biomolecules present in the bioresource, such as sugars, polyphenols, flavonoids, proteins, or enzymes. Subsequently, these atoms assemble to form the AgNPs. Finally, the interaction of these nano-objects with biomolecules via weak or strong interactions determines the quality of their colloidal stability [74,75].

The extracellular pathway is another prominent approach used to synthesize AgNPs in the presence of living cells of bacteria, fungi, and microalgae, although the process itself occurs outside the cells in the culture medium [77]. In this approach, the experimental conditions play a crucial role, such as using different cell types under given growth conditions [53,78,79,80]. In a similar way to the cell-free pathway, the AgNP shape, size and colloidal stability are also influenced by several factors, such as the pH, temperature, and capping agents that are present [67,81,82]. The synthesis follows the different steps described for the extracellular pathway via a similar mechanism, although some specific nitrate-dependent reductases and shuttle quinones might be involved [83,84].

The intracellular synthesis of inorganic NPs constitutes the last methodology followed in the biosynthesis of nanomaterials, in general, and AgNPs in particular [47,85,86]. Compared to the two previous ones, this remains the least explored pathway. Although experimental conditions play a crucial role in this process, the physiology and viability of the living organisms restrict the extent to which these parameters, especially the pH, temperature, and precursor concentration, can be varied. Instead, the focus is usually placed on the employed microorganism, culture age, cell density, and reaction time [87]. Under certain conditions, the intracellular pathway allows the design of bioreactors for the continuous synthesis of NPs [88]. In addition, the very vast majority of studies report on the intracellular synthesis of noble metal, zero-valent NPs [47,86,89,90]. In a few instances, alloy metallic NPs are also obtained [91].

Several studies have explored the use of various bioresources for the intracellular synthesis of AgNPs, including microalgae, plants, mammalian cells and, exceptionally, animals [47]. Importantly, maintaining the cells in their growth media yields results that differ significantly from using either washed cells or cell-free supernatant, affecting many aspects, such as NP size, shape, production kinetics, and colloidal stability [55]. Several species of microalgae and cyanobacteria have demonstrated their ability to effectively synthesize AgNPs via an intracellular pathway, as evidenced by optical and electron microscopy [86,89,92]. On the other hand, whole cultures of the microalga *C. reinhardtii* promote the production of AgNPs simultaneously via extra- and intra-cellular pathways [55]. Although the contribution of each pathway is not quantified, this study clearly shows that the use of the cells in their original medium without any further processing gives the best results in terms of NP features (size, shape, stability, and yield). Other organisms, such as bacteria and fungi, have also potential in the intracellular synthesis of AgNPs [56,93].

## 3. Toxicity of Silver Nanoparticles

A great number of studies have been conducted to assess the toxicity of AgNPs [94,95,96]. Although NPs have several extraordinary features, not all of them are fit to achieve a proper function, thereby causing side effects in living organisms [97]. As NPs have been gaining increasing applications in several fields, such as electronics, energy and healthcare, to name a few, their toxicity has become a booming investigation field due to their impact on human health and the environment [98,99,100]. Several studies have found that the size, shape, surface charge, chemical composition, solubility, dose, exposure route, metabolism and excretion affect the toxicity and biokinetics of NPs [94,95,101,102,103,104,105,106,107,108]. The physical size-dependent characteristics of these materials contribute to hindering metabolism and excretion, provoking a long duration inside the host and consequently a prolonged harmful effect [109].

The oligodynamic effect of metals, especially heavy metals including silver, occurs even when their concentrations are low. The size of AgNPs controls their properties and, thus, their activity towards many species of bacteria [110,111]. The exact mechanism of action of silver NPs on the cell is yet to be fully unraveled [112]. However, a significant amount of data accumulated in this area indicates that AgNPs can physically interact with the cell surface of different microorganisms [82,113,114]. Several observations support this statement regarding NP adhesion to the cell wall and membrane of bacteria: penetration into the cell and disruption of intracellular organelles, induction of oxidative stress, and modulation of signal transduction pathways, among others [101,115,116]. In addition, AgNPs can putatively modulate cellular signaling by dephosphorylating tyrosine residues on key peptide substrates of bacteria, thus inhibiting their growth [30,117].

It is well-known that silver ions do not exert the same toxic effects as AgNPs, mainly because the former are more reactive [118]. The cytotoxicity of AgNPs begins with the significant release of toxic ions that follows their internalization [105]. Since silver ions play a key role in the toxicity of AgNP formulations due to carry-over, their amount should be frequently measured and reported [95]. However, recent findings using mammalian cells indicate that AgNP-induced toxicity might be an intrinsic effect of AgNPs that is independent of free Ag^+^ and the mode of action of AgNPs may differ from that of Ag^+^ since the latter increases H_2_O_2_ driving the oxidative stress and the apoptotic pathways while the former provoke lipid peroxidation causing proteotoxicity and necrotic pathway activation [119]. Furthermore, AgNPs capped with starch and bovine serum albumin (BSA) induce a dose-dependent toxicity in zebrafish embryos and prevent their normal development by exhibiting phenotypic defects and altered physiological functions, such as bradycardia, axial curvatures and degeneration of body parts [120].

The shape of AgNPs is another parameter that significantly affects their toxicity in environmental models [121]. Compared to quasi-spherical AgNPs and silver nanowires, Ag nanocubes exhibit a lower toxicity toward several environmental models, including the ryegrass *Lolium multiflorum*, the zebrafish *Danio rerio*, the nematode *Caenorhabditis elegans*, and bacterial species (*Escherichia coli*, *Bacillus cereus*, and *P. aeruginosa*) [122]. The surface functionalization and charge, temperature and nature of the immersion medium, including the presence of biomolecules and salts, are also major factors that affect the toxicity of AgNPs [123]. In this vein, the physical interactions between AgNPs and *Bacillus* sp. are mainly governed by the surface charge, which has a greater influence on the toxicity than the particle shape and size [124].

**Figure 3:**
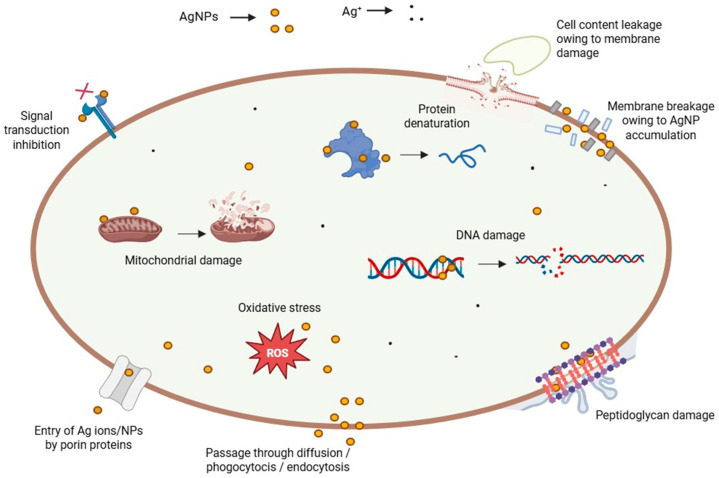
Different toxicity pathways of AgNPs.

Some of the principal toxic effects of AgNPs in mammals are changes induced in the treated cells, including alteration of DNA, oxidative stress, and protein denaturation, among others (Figure 3) [96]. By affecting the dehydrogenase activity, reactive oxygen species (ROS) damage the mitochondria, resulting in diminished ATP production [105]. Consequently, the cell metabolism decreases, and vital functions dramatically plummet or even stop. DNA is also damaged by oxidative stress, which disturbs the correct process of the cell cycle during G2 and M phases [125]. Therefore, the cells undergo apoptosis or necrosis. In addition, the cells can present several chromosomal abnormalities. These toxic effects are concentration-dependent, thus an excessive NP dose during the exposure/treatment can increase the side effects and hurt the patient at a bigger scale [126]. Other related cytotoxic effects stem from the interference of NPs with specialized proteins, such as the membrane protein links and enzymes, like lactate dehydrogenase (LDH) [103]. In addition, peptidoglycans, which are an exclusive structural feature of bacteria, constitute the critical reason why AgNPs are highly toxic to these prokaryotes when compared to eukaryotic cells. AgNP interaction with bacteria leads to bacterial membrane damage, as observed by TEM and SEM. The membrane damage was confirmed by detecting the leakage of proteins and reducing sugars from treated bacterial cells [114,127]. Conversely, the protein corona that builds up on biogenic AgNPs appears to be responsible not only for making them more stable in time but also for masking and protecting eukaryotic cells against metal toxicity [128], thus explaining, at least in part, the term “paradoxical” in this review’s title.

Several studies discuss the toxicological effects of *bio*-AgNPs. For instance, *bio*-AgNPs prepared with the aqueous leaf extract of *Swertia chirata* are equally toxic to *Allium cepa* cells as chemically synthesized AgNPs and silver ions [129]. Additionally, the induced chromosomal aberrations are similar both at mitotic and meiotic levels even at lower concentrations. Rheder et al. (2018) show that *bio*-AgNPs synthesized by the leaf extract of the plant *Althaea officinalis* (AgNP-L) are slightly more toxic towards the used mammalian cell lines than those synthesized with the dehydrated root infusion of the same plant (AgNP-R); this might be related most likely to a size effect since the former are smaller than the latter [130]. This trend is also observed when these two types of *bio*-AgNPs are tested on zebrafish. Whereas the two highest tested concentrations lead to fish death after 24 h exposure, the intermediate concentration causes death of fish exposed to AgNP-L and great damage to the gill cells in fish exposed to AgNP-R, while the lowest provokes DNA damage in blood cells, regardless of NP type. Furthermore, high concentrations of *bio*-AgNPs made using *Rumex acetosa* inhibit the proliferation of human umbilical vein endothelial cells (HUVECs) via a ROS-induced apoptotic pathway and cause morphological changes in the yolk sac and the tail of zebrafish [131].

## 4. Biological Properties of *bio*-AgNPs

**Figure 4:**
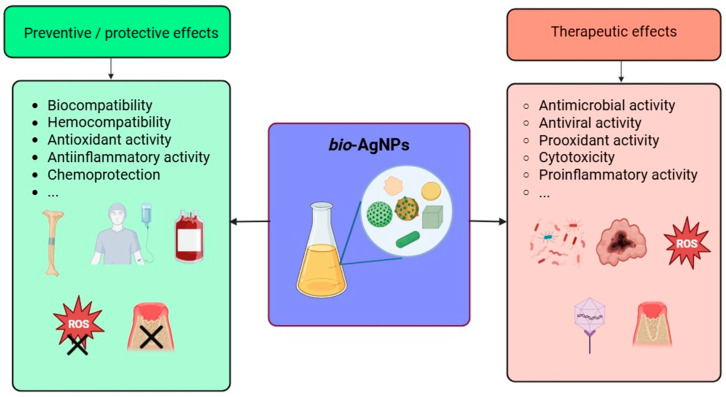
Illustration of some of the most noteworthy biological properties of biogenic AgNPs.

Inorganic NPs display a very diverse and rich set of biological properties that make them of paramount importance in various applications related to human health and well-being, such as the biomedical field, agriculture, and the environment (see above). As portrayed in Figure 4, these outstanding properties encompass but are not limited to anti- and pro-oxidant activity, anti- and pro-inflammatory activity, modulation of the immune system response, inhibition of enzymatic pathways, induction of apoptosis/necrosis, and, depending on the target, genotoxicity, cytotoxicity against pathogens and cancer cells, and biocompatibility for healthy cells and tissues, enabling their use in several biomedical applications (cf. next section). These properties, present among inorganic NPs that are obtained via a green approach, might even be enhanced due to the moieties of biological origin, i.e., biomolecules, phytochemicals, that coat their surface [132,133,134]. For instance, biogenic selenium NPs exhibit valuable characteristics in addition to the abovementioned properties since they also lessen the toxicity caused by drugs or heavy metal cations [135]. Similarly, *bio*-AgNPs are also known to possess unique biological properties and activities that are described in the following paragraphs.

The oxidative stress refers to the accumulation of reactive oxygen species (ROS) in cells and tissues because of an imbalance between their production and detoxification [136]. The excess and accumulation of ROS may lead to the onset of numerous diseases, including cancer, diabetes, Alzheimer’s, and atherosclerosis [137]. Oxidative stress is usually studied in vitro using several routine assays, such as 2′,7′-dichlorofluorescein diacetate (DCFH-DA) [138,139], 2,2′-diphenyl-1-picrylhydrazyl radical (DPPH) [140,141], 2,2′-azinobis-(3-ethylbenzothiazoline-6-sulfonate) (ABTS) [142,143], ferric reduction antioxidant power (FRAP) [143,144], and phosphomolybdenum assays [145].

The ability of NPs to trigger the cellular production of ROS is known as the prooxidant activity. On the other hand, the scavenging of these radicals by the NPs defines the antioxidant activity. In this regard, *bio*-AgNPs are outstandingly versatile since they can either provoke a surge in the intracellular ROS levels, which make them toxic to pathogenic microbes, such as bacteria [146,147,148], fungi [149,150,151], and viruses [152], in addition to their cytotoxicity against countless cancer cell lines [153,154]. On the other hand, *bio*-AgNPs can lower the ROS levels, rendering them protective of healthy cells and organs [155,156,157]. These findings constitute a special feature of biologically synthesized NPs. However, *bio*-AgNPs hold a distinct position because of the ease and speed of their synthesis which is carried out, usually, under straightforward conditions: simple glassware, atmospheric pressure, room temperature, or very mild heating, either in the dark or under regular illumination, no pH adjustment, etc., (see above).

These oxidative radicals possess several modes of action against pathogens and cancer cells: they may (i) damage the cellular integrity by perforating the cytoplasmic membrane and cause the leakage of different components in bacteria [158,159,160,161]; (ii) induce DNA damage [158]; (iii) induce autophagy and apoptosis in cancer cells [138,140,150,162,163]; (iv) upregulate pro-apoptotic pathways and/or downregulate their anti-apoptotic analogs [164]. On the other hand, *bio*-AgNPs scavenge the ROS and, therefore, prevent their harmful effects against healthy cells [165]. This remarkable activity explains the surge in the exploration of the opportunities that *bio*-AgNPs might offer in combating infections and emerging threats to humans and their environment (see below).

Several studies underline the major role that *bio*-AgNPs may play in the modulation of inflammation in humans since they can favor the body’s defense mechanisms, yielding a pro-inflammatory response, or, on the contrary, fight against it via an anti-inflammatory pathway [166,167,168,169,170]. On a molecular level, *bio*-AgNPs decrease mRNA levels of inflammation-related enzymes and pro-inflammatory cytokines in lipopolysaccharide-stimulated RAW 264.7 cells [168], scavenge the activity of nitrite [143,171], attenuate the activity of inducible nitric oxide synthase (iNOS) and cyclooxygenase-2 (COX-2) [172], and prostaglandin E2 [171], lower the level of pro-inflammatory cytokines [167,172,173,174], promote the expression of anti-inflammatory cytokines [167], and inhibit the activity of proteinases [175]. Moreover, *bio*-AgNPs can reduce the heat-induced effect on bovine serum albumin (BSA) [176], greatly inhibit protein denaturation, and stabilize the membrane of human red blood cells [142,148,176,177,178,179]. In animal models, *bio*-AgNPs suppress pro-inflammatory cytokines, such as TNF-α, IL-1β, and IL-6 [167,174,180], and promote the expression of the anti-inflammatory cytokine IL-10 on burn injury [167,174]. The modulation of the inflammatory response appears to be dose-dependent in xenograft-bearing mice [156]. In vivo experiments on mice bearing acute myeloid leukemia demonstrate clearly that *bio*-AgNPs lower the total count of the different leukocytes, favor body weight gain, and restore the lymphocyte, platelet, and RBC counts [174]. On a macroscopic level, *bio*-AgNPs have proven efficacious in reducing the volume of induced edema or preventing its onset in treated mice [180].

Other studies clearly demonstrate the ability of *bio*-AgNPs to inhibit the activity of several enzymes. For instance, the capacity to inhibit the activity of several metabolic enzymes, such as α-amylase, α-glycosidase, and dipeptidyl peptidase IV, confers these *bio*-NPs a valuable antidiabetic character [142,148,176,181]. *bio*-AgNPs can also inhibit the action of enzymes related to aging processes [182,183], in addition to others involved in the onset of Alzheimer’s disease [175,183] and in other biological processes [148,183,184].

Lastly, several in vitro and in vivo studies highlight the biocompatibility of *bio*-AgNPs. These findings are of crucial importance, especially from the point of view of developing *bio*-AgNP-based nanoformulations and devices for real-world applications related to human health and well-being. For instance, *bio*-AgNPs do not induce any hemolysis [164,185,186,187] and possess valuable thrombolytic activity [146,188,189]. *bio*-AgNPs seem not to interact with hemoglobin and human serum albumin [190], although another study reports that *bio*-AgNPs bind to the latter, resulting in a slight modification of its structure [191]. Other studies pinpoint the biocompatibility of *bio*-AgNPs towards, for instance, human peripheral blood lymphocytes [192] and fibroblasts [187,193,194]. These findings are corroborated by in vivo tests that reveal no noticeable toxicity of *bio*-AgNPs administered orally [195,196], intravenously [197], or intraperitoneally [198] in healthy mice. On the other hand, *bio*-AgNPs do not cause any major toxicity when used to treat tumor-bearing mice, and they might also protect these animal models against the toxicity induced by the conventional drugs used (see below).

## 5. Bioapplications of bio-AgNPs

**Figure 5:**
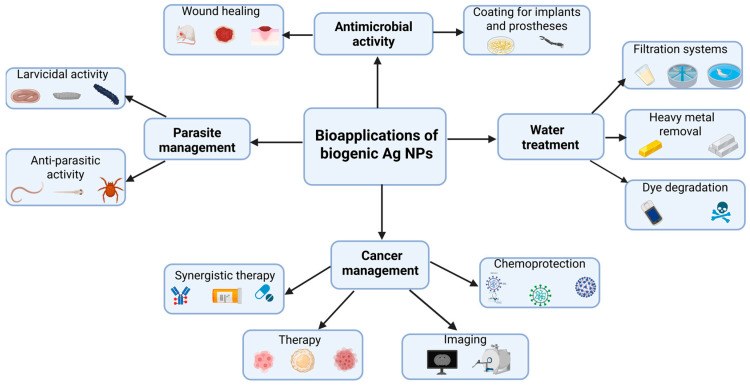
Summary of the most widespread bioapplications of biogenic AgNPs.

Owing to their outstanding biological properties, *bio*-AgNPs have been explored for numerous applications in the biomedical field and water treatment (Figure 5). The following paragraphs will provide insightful discussion on each of them with examples taken from recently published findings.

### 5.1. Larvicidal Activity of bio-AgNPs

In the era of climate change and globalization, the control of vectors of dangerous diseases, such as mosquitoes, that transmit yellow fever, chikungunya fever, dengue hemorrhagic fever, malaria and filariasis, among others, is a major worldwide concern for health authorities [199,200]. As a green alternative to commercially available pesticides, much attention has been placed on eco-friendly yet cost-effective solutions for the control of proliferation of these insects thanks to the larvicidal efficacy of phytocompounds, plant extracts and plant-based metallic NPs [201,202,203]. Although still laboratory-based, these emerging approaches bring hope for field applications as they take advantage of the intrinsic toxicity of inorganic nanomaterials to destroy the colonies of mosquito eggs, larvae, and pupae [204,205]. Several studies describe the use of *bio*-AgNPs as a potent larvicidal agent [206,207,208]. For instance, *bio*-AgNPs produced using the leaf aqueous extract of *Aegle marmelos* exhibit a dose-dependent larvicidal activity against larvae of both *Aedes aegypti* and *Culex quinquefasciatus,* where the lethal concentration that kills 50% of the larvae, LC_50_, is much lower in the latter (132 ppm) than in the former (302 ppm) [209]. These findings corroborate other results that demonstrate the killing efficacy of *bio*-AgNPs, synthesized using other plant-based extracts, such as those originating from roots [210], fibers [211], stems, leaves [212], bark [213], fruits [214], latex [215], and shoots [216] against the larvae of several mosquitoes that are vectors of diseases. Likewise, *bio*-AgNPs of fungal or bacterial origin, are also a powerful weapon that destroys mosquito larvae in a dose-dependent manner [217,218,219,220,221,222].

Several experimental parameters controlling the larvicidal activity of *bio*-AgNPs have been explored, such as the applied dose, the exposure duration, development stage, and target species. Several studies have reported the dose-dependent action of *bio*-AgNPs against the larvae, regardless of the type of biomass used or the nature of the extract [219,220,221]. Usually, the higher the dose, the higher the larvicidal activity [217,223]. Sometimes, *bio*-AgNPs synthesized using a given biomass exhibit better larvicidal activity than the same dose of *bio*-AgNPs obtained through a different type of biomass. For instance, more than 33 ppm of *bio*-AgNPs, produced using an aqueous extract of *B. amyloliquefaciens*, are needed to reach the LC_50_ against the pupae of *C. pipiens pallens,* while less than 14 ppm are needed for the same goal when *B. subtilis* is used instead [222]. Exposure duration is another parameter that impacts the larvicidal activity [211,224]. For instance, a given dose of *bio*-AgNPs applied against *A. aegypti* larvae has a higher activity after 48 h of exposure when compared to an exposure of 24 h, although the difference tends to diminish when the applied dose increases [210]. Several studies shed light on the larvicidal activity of *bio*-AgNPs as a function of the development stage of the larvae [222,225,226]. Murugan et al. report that, for all screened doses of *bio*-AgNPs, the mortality of *C. quinquefasciatus* decreases as it progresses in its development, from instars I to IV as larvae to pupae [227]. A similar trend is reported regarding the mortality of larval instars of the same vector and of *A. aegypti* [221]. In other words, destroying larvae colonies requires higher doses of *bio*-AgNPs as the mosquitoes develop. However, instar II is reported to be more susceptible to *bio*-AgNPs when compared to the other stages since they show a 100% mortality within 1 h exposure [219]. Further, the same group demonstrated that *bio*-AgNPs are more efficient than their Au counterparts synthesized using the same *Chrysosporium tropicum* fungal supernatant against the same vector. Divergent trends have also been reported, i.e., increased % mortality for all screened concentrations of *bio*-AgNPs as the larvae develop [228], but with no adequate explanation.

Some studies compare the larvicidal activity inherent in the extract used to synthesize the *bio*-AgNPs with that of the resulting NPs [207]. For instance, it is reported that the concentration, in μg/mL, of the extract, prepared from the shoots of *Echinochloa stagnina*, should be multiplied by ~5–7 fold to reach the same larvicidal activity (LC_50_ and LC_90_) against *Anopheles pharoensis* and *C. pipiens* as that of the resulting *bio*-AgNPs [216]. To reach the same mortality effect on larvae at their II and IV instars, it has been shown that the dose in ppm of the aqueous crude latex should be multiplied by two orders of magnitude when compared to the effect of the resulting NPs [215]. As a general trend, the larvicidal activity of *bio*-AgNPs is always stronger than that of the biomass extract used for their formation [212,229,230,231]. However, the biomass itself seems to play a non-negligible role in the larvicidal activity. For instance, *bio*-AgNPs synthesized using clove aqueous extract exhibit a much higher and faster larvicidal activity than their analogs obtained using NaBH_4_ or glutathione; this effect might arise from the phytochemicals present in the extract [232]. This reinforces previous observations that the increase in polyphenols enhances the larvicidal activity of *bio*-AgNPs against *Spodoptera littoralis* larvae [233].

Several groups have attempted to unravel the mechanistic aspects underlying the larvicidal activity of *bio*-AgNPs and its specificity. Owing to their nanoscale size, AgNPs may cross the insect cuticle and penetrate the cells where they interfere with physiological processes, including molting [204]. When challenged by *bio*-AgNPs, IV^th^ instars of *C. pipiens pallens* undergo morphological changes in their thorax and abdomen, resulting in significant damage in the anal region and cuticle layer [222]. On the other hand, the pupae of the same mosquito exhibit, after exposure to *bio*-AgNPs, severe distortions in the head, thorax and abdomen, and loss of breathing ability. Besides confirming the shrinkage of the internal cuticle, another study describes the pigmentation and swelling of apical cells of *A. aegypti* larvae after exposure to *bio*-AgNPs [234]. These findings highlight the disturbances induced by *bio*-AgNPs upon the normal development of the larvae. The uptake of *bio*-AgNPs might be facilitated by the biomolecules present in the synthesizing biomass extract since some of these moieties bind specifically to mosquito salivary proteins [235]. Histopathological images of *bio*-AgNP-treated larvae of several mosquito species display, among other things, altered structures of the midgut epithelial cells, apical enlargement in the gut lumen, and reduction in intercellular connections [220].

The above-mentioned studies underscore the strong larvicidal activity of *bio*-AgNPs when compared to their chemical analogs or to the extracts used in the synthesis process. However, there are concerns about the potential toxicity of these NPs should they be used on a large scale and translated from the lab bench to the field in the form of *bio*-AgNP-based commercial nanopesticides to combat the development of mosquito larvae responsible for spreading diseases to humans and livestock [207]. Further work is needed to address these issues by enabling the design of long-term safe *bio*-AgNP nanoformulations that are suitable for field use and by fully elucidating their mode of action. A handful of studies offer optimistic perspectives in this area since, paradoxically, *bio*-AgNPs do not show toxicity towards non-target species [230,236,237,238]. Other studies show that *bio*-AgNPs increase predation against the NP-treated larvae [226,239]. However, the long-term fate of released *bio*-AgNPs in the environment and their impact on the ecosystems should be meticulously monitored.

### 5.2. Antiparasitic Activity of bio-AgNPs

Parasite-borne illnesses affect humans and animals and cause mild-to-severe health problems that can lead to important economic and social consequences and, sometimes, to death [199]. For instance, leishmaniasis, the most widespread parasitic infection, is caused by the protozoans belonging to the *Leishmania* family, which are transmitted by the bite of infected female phlebotomine sandflies. Leishmaniasis affects between 700,000 and 1.2 million persons living in some of the poorest countries in the world; it has different clinical forms, the cutaneous, mucocutaneous, and visceral among which the latter is life-threatening if left untreated [240]. Despite the commercial availability of several antiparasitic drugs [241], some of them have disadvantages, such as their cost, given that these infections affect very poor populations, their toxicity, and the resistance some parasite carriers can acquire, rendering the therapeutic molecules progressively inefficient and useless [242]. In addition to conventional therapies, several groups have questioned the capabilities offered by nanotechnology in managing these infections [241,243,244,245]. Typically, several NPs of different provenance, such as those made of polymers, fibers, lipids, and metals, have been explored for their ability to inhibit the growth of these parasites and/or their activity, and induce lethal damage [241,246,247,248]. Several parasite-borne diseases have been investigated for potential treatment using *bio*-AgNPs, including malaria caused by *Plasmodium* spp. [249], leishmaniasis caused *Leishmania* spp. [250], trypanosomiasis (Chagas Disease and African Sleeping Sickness) caused by *Trypanosoma cruzi* [251] and *T. brucei* [252], schistosomiasis caused by *Schistosoma* spp. [253], toxoplasmosis caused by *Toxoplasma gondii* [254] and giardiasis caused by *Giardia lamblia* [255].

Using representative examples, the following paragraphs portray the activity of *bio*-AgNPs against different parasites causing infections that primarily impact humans, especially leishmaniasis which remains, by far, the most targeted infection of parasitic origin [244,256]. In vitro studies show that chemically synthesized NPs made of noble metals (Au, Ag and Pt) and their alloys restrict the growth of *Leishmania tropica*, *Toxoplasma gondii* and different *Trypanosoma* strains in a dose-dependent manner [252,257,258]. In the case of *T. gondii*, the best results, in terms of, for instance, parasite viability, parasite invasion rate, and parasite intracellular replication, are obtained with *bio*-AgNPs whereas the viability of host cells remains quite unaffected [258]. The action of these NPs against *T. gondii* might arise from the generation of ROS. It is also possible to enhance the antiparasitic activity of *chem*-AgNPs consequently to ultraviolet light irradiation [257].

*bio*-AgNPs exhibit a versatile and appreciable in vitro antiparasitic activity against several pathogens, such as *L. amazonensis* [259,260], *L. donovani* [261], *L. major* [262], *L. tropica* [263], *Plasmodium falciparum* [264,265], *Giardia lamblia* [255], *Pythium insidiosum* [266], *Trypanosoma brucei gambiense* [267,268], *Schistosomiasis mansoni* [253], *T. gondii* [254]. Interestingly, the same *bio*-AgNPs exert mild to negligible cytotoxic effects on mammalian cells showcasing, thus, their biocompatibility. In addition, *bio*-AgNPs inhibit the growth of *L. major* at both the promastigote and amastigote stages [269]. Moreover, *bio*-AgNPs possess an appreciable anthelmintic activity since those produced using the aqueous extract of *Lansium parasiticum* exhibit toxicity towards adult males and females, larvae, and eggs of *Haemonchus contortus*, a nematode causing infection in the gastrointestinal tract [270]. Furthermore, *bio*-AgNPs greatly enhance the antiparasitic activity of drugs, such as miltefosine [271]. However, in some cases *bio*-AgNPs are less (or equally) efficacious than a drug, e.g., pyrimethamine [272]. Lastly, *bio*-AgNPs obtained using the leaf extract of the medicinal plant *Teucrium stocksianum* possess a better antiparasitic activity against promastigotes of *L. infantum* than the ones made using the stem extract of the same plant although both show a dose-dependent activity [273].

The aqueous extract of the medicinal plant *Sargentodoxa cuneata* enables the production of *bio*-AgNPs that are lethal to *L. tropica* in a dose- and time-dependent manner since the viability of the parasite nearly collapses to zero while its growth is fully inhibited within 24 h of exposure [274]. At the same time, *bio*-AgNPs show the best results when compared to the extract alone or to *bio*-AuNPs obtained using the same extract. In vitro studies show that *bio*-AgNPs, obtained using the medicinal herb myrrh or *Commiphora molmol*, have the best sustained effect against *L. major* when compared to chemical analogs or the drug pentostam [275]. At the same time, the area of cutaneous lesion due to leishmaniasis in murine models recedes faster when *bio*-AgNPs are applied topically than when relying on *chem*-AgNPs or the drug pentostam [275]. Furthermore, *bio*-AgNPs appear to have no adverse effects on mice kidneys and livers. These findings corroborate the results reported earlier [259]. On the other hand, *bio*-AgNPs appear to enhance the therapeutic effect of amphotericin B against *L. tropica* since, in all tested concentrations, and the combined formulation yields a better effect enabling thus to lower the concentration of used silver; however, there is no mention of the sole effect of amphotericin B [276]. Likewise, ointments containing both *bio*-AgNPs and quercetin show a dramatic effect in healing leishmaniasis-induced ulcer in mice [277]. The same report also records an IC_50_ of 6.125 µg/mL for *bio*-AgNPs that should be compared to 100 µg/mL for the leaf extract of *Artemisia aucheri* used to synthesize the *bio*-AgNPs, and to 150 µg/mL for quercetin, thus demonstrating the antileishmanial efficacy of *bio*-AgNPs. Another in vivo study demonstrates the positive impact of *bio*-AgNPs in healing mice infected with schistosomiasis, especially when combined with the drug praziquantel [278]. From a mechanistic point of view, there is a lot to discover as various pathways might be involved [244]. A few studies clearly highlight the dependence of the results on the targeted parasite, infected cell lines and origin of *bio*-AgNPs [279]. Overall, *bio*-AgNPs hold great promise in managing parasitic infections, such as leishmaniasis, especially when used as part of topical formulations that might facilitate translational knowledge from animal models to humans [248,277].

### 5.3. Biogenic AgNPs as a Promising Tool in Cancer Imaging and Therapy

Owing to their unique properties, some of which are discussed above, *bio*-AgNPs exhibit a very large spectrum of activity against various cancer cell lines and experimental tumor xenografts [280,281]. Several articles extensively review the in vitro anticancer activity of *bio*-AgNPs against malignant cell lines, highlighting their quite universal cytotoxic (or antiproliferative) propensity as encountered among all tested NPs [282]. Therefore, the present section aims to sum up the most important, and updated findings regarding this aspect by portraying the richness and diversity of the potential of *bio*-AgNPs in cancer therapy. The biomass used for their fabrication may originate from different sources, such as plants [283,284], algae [285,286], bacteria [139,287,288,289], and fungi [290,291,292]. Both officinal [293,294,295,296] and non-officinal plants [142,297,298] are used to produce *bio*-AgNPs possessing an in vitro anticancer activity against, mostly, human cancer cell lines, and, in a few cases, murine cancer lines [149,174,299]. To reach the same goal, different parts of plants might be exploited, such as sea grass [300], leaves [301], stems [302], flowers [303], fruits [304,305], seeds [306], spices [307], roots [308], or specific biomolecules isolated from plants [164]. Although most studies detail the utilization of aqueous extracts in the biosynthesis of *bio*-AgNPs, a few others report the use of ethanolic extracts [309,310,311]. Whenever the extraction occurs using organic solvents, such as ethanol, the resulting solvent-free extract is transferred into water [312].

The in vitro anticancer activity of *bio*-AgNPs is dose-dependent since the cell viability decreases with increasing NP concentration [313,314,315]. It is also time-dependent [309,316]. Most often, *bio*-AgNPs exhibit a superior anticancer activity when compared to that of the extract itself [177,311,317,318,319]. However, in a few instances, no significant difference in the anticancer effect is observed when comparing *bio*-AgNPs with the aqueous extract itself [291,320]. So far, there is a lack of information regarding the impact of the size and shape of *bio*-AgNPs on their in vitro anticancer activity. When compared to other NPs synthesized via the same green process, i.e., same plant extract and protocol, such as *bio*-AuNPs, no clear trend emerges as contradictory data is reported; this might be explained by the variation in NP size and shape and the response of the tested cells [320,321,322]. However, *bio*-AgNPs seem to be more potent than cationic silver administered at the same concentration [323,324]. On the other hand, *bio*-AgNPs remain, by far, more powerful in inhibiting the in vitro growth of cancer cells, while at the same time remaining more biocompatible than their chemical analogs [193,313]. This extra anticancer effect might be attributable to the biopolymeric layer, rich in bioactive biomolecules, like terpenoids, saponins, flavonoids, and phenols, that surround the NPs [302,325,326]. The polymeric matrix may also foster a better binding between the *bio*-AgNPs and their target cells, thus enhancing the anticancer efficacy of the NPs [327].

When compared to conventional cancer drugs, there are no clear data that show any in vitro superiority of *bio*-AgNPs, since a few studies report contradictory results [328,329]. This might be attributed to the interplay of several parameters, including the NP features (size, shape, coating), the cell line, and the drug used. *bio*-AgNPs might also be used to enhance the efficacy of conventional chemotherapy drugs, e.g., cisplatin, while, at the same time, reducing their side-effects on healthy body tissues and organs [155,156,329]. Some studies pinpoint the fact that *bio*-AgNPs are toxic to cancer cell lines while they remain innocuous to healthy ones [330,331,332]. This is corroborated by other findings suggesting a discriminatory lethal action of *bio*-AgNPs against cancer cells while they remain biocompatible when tested on healthy cell lines [333]. This most likely originates from the NP coating made of the bioresource extract used. However, further investigations are needed to elucidate this fact.

It is possible to encapsulate the *bio*-AgNPs in another matrix made of chitosan, for instance; however, this does not always ensure the newly designed AgNP-based formulation any substantial advantages over the other AgNP-free formulations in terms of scavenging activity or cytotoxic effects on cancer cells [334]. In other instances, the post-functionalization greatly improves the biocompatibility of the *bio*-AgNPs without affecting their anticancer efficacy [299].

Several groups have investigated the mode of action of *bio*-AgNPs against various cancer cell lines [335,336,337,338,339,340]. This action may result in morphological alterations, DNA fragmentation, ROS generation, impairment of mitochondria function, gene and protein down-/up-regulation, and, ultimately, induction of apoptotic pathways [153,280,321,341,342,343,344]. For instance, *bio*-AgNPs, produced using the marine alga *Chaetomorpha linum*, act via an apoptotic pathway since they increase, on one hand, the expression of apoptotic proteins, such as caspase 3 and 9, and Bax, while, on the other hand, they decrease the expression of the anti-apoptotic proteins Bcl-2 and Bcl-xl, yielding, among other effects, mitochondrial dysfunction that results in the apoptosis of the *bio*-AgNP-treated HCT-116 cancer cell line [324]. Several studies corroborate these findings [303,344]. Besides clearly showing their modulation of gene expression, other studies reveal DNA fragmentation and nucleus condensation, thus, highlighting an apoptotic cell death as a consequence of *bio*-AgNP exposure [312,345]. Flow cytometry analyses indicate that the increase in *bio*-AgNP concentration impacts the viability of cancer cells as the proportion of dying cells increases with a clear shift towards late apoptosis and necrosis [346]. Moreover, *bio*-AgNPs downregulate the oncogenes PIK3Ca and KRAS [347]. The exposure to *bio*-AgNPs eventually leads to apoptosis as it triggers, in a dose-dependent manner, the impairment of cellular membranes and increased lactate dehydrogenase leakage; it also impairs the mitochondrial function indicated, for instance, by greater levels of ROS and malondialdehyde, when compared to untreated controls [314]. Interestingly, genes related to oxidation-reduction pathways are upregulated, especially the ones coding for cytochrome P450 monooxygenases; on the other hand, genes linked to aging are upregulated [314]. *bio*-AgNPs, obtained using the aqueous leaf extract of *Eucalyptus globulus*, are also able to inhibit the formation of cancer cell colonies [332].

Besides their intrinsic, rich biological activity, *bio*-AgNPs may also be used as a radiosensitizing agent that absorbs γ-rays [348]. Indeed, a 6-Gy dose of γ-rays reduced the viability of HepG-2 cells treated with *bio*-AgNPs synthesized using the leaf aqueous extract of *Picrasma quassioides*, by more than 90%. These findings may suggest the accumulation of these *bio*-AgNPs in the vicinity of the cells and/or their internalization owing to a passive accumulation process since no targeting functionalization is carried out. The in vivo anticancer activity is also found in biogenic NPs that are made of a mixture of silver and silver chloride [349,350]. On a cellular level, these NPs exert their toxicity by altering the expression of genes that eventually leads to apoptosis.

Several groups have taken a step forward by investigating the efficacy of *bio*-AgNPs in combating solid tumors in animal models. Bacterially synthesized *bio*-AgNPs were used at 500 nM via intraperitoneal (IP) injection for 15 days to monitor their effect on solid xenograft in female Swiss albino mice in addition to a thorough in vitro investigation [288]. First, it was confirmed that these purified, endotoxin-free *bio*-AgNPs had no adverse side effects on healthy mice, thus highlighting their biocompatibility. These findings were later corroborated by other studies [196,197,198,351]. Second, mice treated with *bio*-AgNPs had a longer survival time when compared to their control counterparts (32 vs. 18 days) and saw their tumor volume dramatically recede by two thirds (2.6 mL vs. 7.3 mL for the control) [288]. Third, only minor alterations in hematological parameters were observed among tumor-bearing mice that were treated with *bio*-AgNPs. This study suggests that *bio*-AgNPs naturally accumulate in tumors without the need for any further post-functionalization step to provide them with stealth and targeting properties. These early findings were corroborated by He al., who used the peel powder of the longan fruit to synthesize *bio*-AgNPs for IP injection in mice settling on 10 µg/g body weight as the working concentration [340]. *bio*-AgNPs slowed down the growth of the tumor and decreased its volume, when compared to the control group. On day 36, the tumor size had receded by more than a half in treated mice. Again, toxicity tests indicate that *bio*-AgNPs efficiently and preferentially target and accumulate in the tumor without any need for post-functionalization. This almost intrinsic and unique feature of *bio*-AgNPs is time-dependent since, for instance, the very weak Raman imaging signal, recorded 5 min after inoculation, becomes extremely strong when taken at 45 min [198].

*bio*-AgNPs can be administered via several modes: intraperitoneally (IP) [288], intratumorally [198], intravesically [352], intravenously [174], and orally [156,353]. Usually, *bio*-AgNPs are quite biocompatible [165,195,197,288,351] and seem to preferentially exert their major cytotoxic effects almost exclusively on cancer cells and tumors via oxidative stress [165,198,353] and/or inhibition of angiogenesis [171]. Therefore, several biochemical parameters, including the activity of specific enzymes, up- and down-regulation of genes, immunological markers, and hematological and cellular parameters, are screened by investigators [155,156,174,353]. Usually, the translation to in vivo studies confirms the up- and down-regulation of genes that are already implicated in in vitro studies [324,353,354]. The in vivo anticancer activity of *bio*-AgNPs is dose-dependent [355]; some biochemical and histological parameters of AgNP-treated tumor-bearing mice may, at first, undergo some changes before reverting to normal values [356].

**Figure 6:**
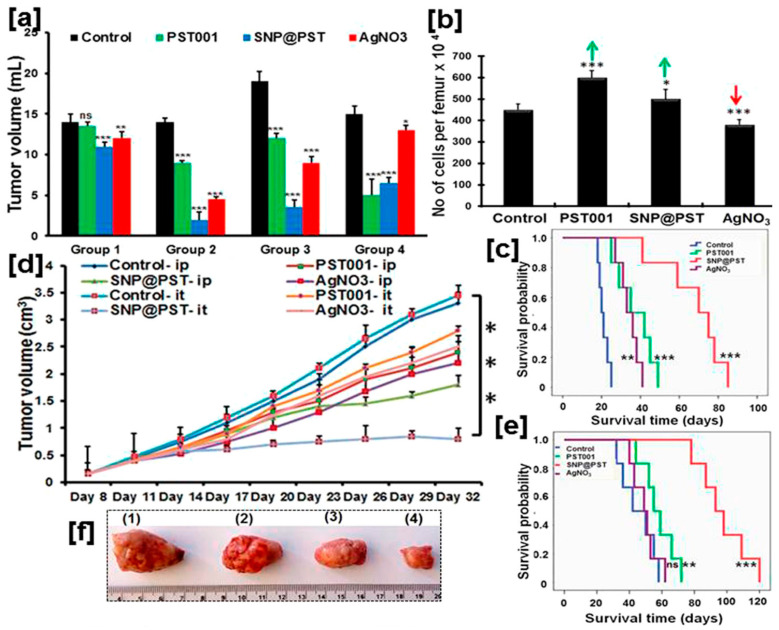
Antineoplastic activity of *bio*-AgNPs synthesized using the lab-purified polysaccharide PST001 galactoxyloglucan (SNP@PST). (**a**) Volume measurements in female BALB/c mice bearing Dalton’s Lymphoma ascites (DLA) xenograft. Groups 1 to 3 are inoculated with DLA on Day 1 while Group 4 is injected on Day 8. Regarding the treatment experiments, Group 1 is treated only once (Day 2), Group 2 is injected on Days 2 to 15, Group 3 is treated on Days 9 to 22, and Group 4 is injected on Days 1 to 7. (**b**) Quantification of bone marrow cellularity in Group 2. Green arrows indicate increase in cell number vs. the control; on the other hand, the red arrow indicates a decrease in cell number vs. the control. (**c**) Survival rate of Group 2 mice bearing DLA xenograft over time as a function of administered compounds. (**d**) Tumor volume measurements in mice bearing Ehrlich ascites carcinoma (EAC)-induced tumor syngraft as a function of received compound and mode of administration (*ip* = intraperitoneal administration; *it* = intratumoral administration). (**e**) Survival rate of Group 3 mice bearing EAC syngraft over time as a function of *it*-injected compounds. For (**c**–**e**), the results are expressed as the mean ± the standard deviation. Statistical significance is denoted as * *p* < 0.05, ** *p* < 0.01, *** *p* < 0.001, and ns (nonsignificant), all compared with the control group. (**f**) Images of resected solid tumors from mice that are *it* treated: (1) Control, (2) PST, (3) AgNO_3_, and (4) SNP@PST. Adapted from Ref. [198] with permission from the American Chemical Society.

Lab-extracted and -purified polysaccharides (PS) are utilized to synthesize AgNPs that exhibit unique anticancer efficacy in animal models [198,357]. For instance, galactoxyloglucan-coated *bio*-AgNPs (PS-AgNPs) do not induce any behavioral changes or noticeable toxicity in healthy male BALB/c mice after administration at a 1.1 µg/g body weight [198]. Biochemical and hematological analyses confirm the absence of any abnormalities for the tested concentrations. Moreover, histological analyses indicate that the vital organs, i.e., heart, kidney, liver, spleen, lungs, remain unaffected by PS-AgNP IP inoculation although some moderate alterations are observed at the highest tested concentration (222 µg/g body weight) [198]. All these findings highlight the biocompatibility of these *bio*-AgNPs that is ensured, most likely, by the coating polysaccharides. When tested on tumor-bearing mice, PS-AgNPs give the best results in terms of all screened parameters: reduction in tumor volume, maintenance of body weight, lowering tumor cell count, diminution of percentage of viable cells, and increase in survival time (Figure 6). In addition, no systemic toxicity is recorded. Owing to their optical properties, the biodistribution of these IP injected PS-AgNPs is monitored in the tumor, blood and vital organs of treated mice using a confocal Raman microscope and found to evolve with time. While the PS-AgNP concentration in blood decreases over time, it increases in the tumor to reach its maximum 4 h after inoculation, then decreases. A similar trend is recorded for the kidney. The amount of PS-AgNPs steadily increases for the screened 6 h period. For the other organs, only tiny amounts of PS-AgNPs are detected after 6 h. Except for kidney and lungs, these results are corroborated by fluorescence analyses [198].

Only a few papers have compared the in vivo anticancer efficacy of bio-AgNPs vs. conventional chemotherapy drugs, such as doxorubicin (DOX) [174,354], and cisplatin (*cis*-Pt) [329]. A few other papers carried out the same work using silymarin, a compound extracted from the plant milk thistle (*Silybum marianum*) that is sold as a dietary supplement and supposed to have an anticancer activity [155,156,327]. When silymarin was used at a 30 mg/g concentration, no statistically significant differences were observed for almost all the screened parameters when compared to doses of 20 or 30 mg/g of *bio*-AgNPs, obtained using the aqueous extract of leaves of the officinal plant *Carissa caranda* [155]. Similar trends were observed when the aqueous extracts of *Madhuca longifolia* [156] or of *Ziziphus mauritiana* were used [327]. Regarding DOX, minor to non-significant differences were reported for the studied hematological, cellular, immunological, and general patient parameters when compared to *bio*-AgNPs [174]. On the contrary, it was found that administered *bio*-AgNPs provide a better protection against the side effects of DOX, owing to the NP antioxidant activity [358]. Importantly, synergistic improvements were obtained by combining DOX and *bio*-AgNPs in terms of enhanced therapeutic effects and better protection against the side effects [354]. Moreover, comparable results were reported using *cis*-Pt instead of DOX [329].

**Figure 7:**
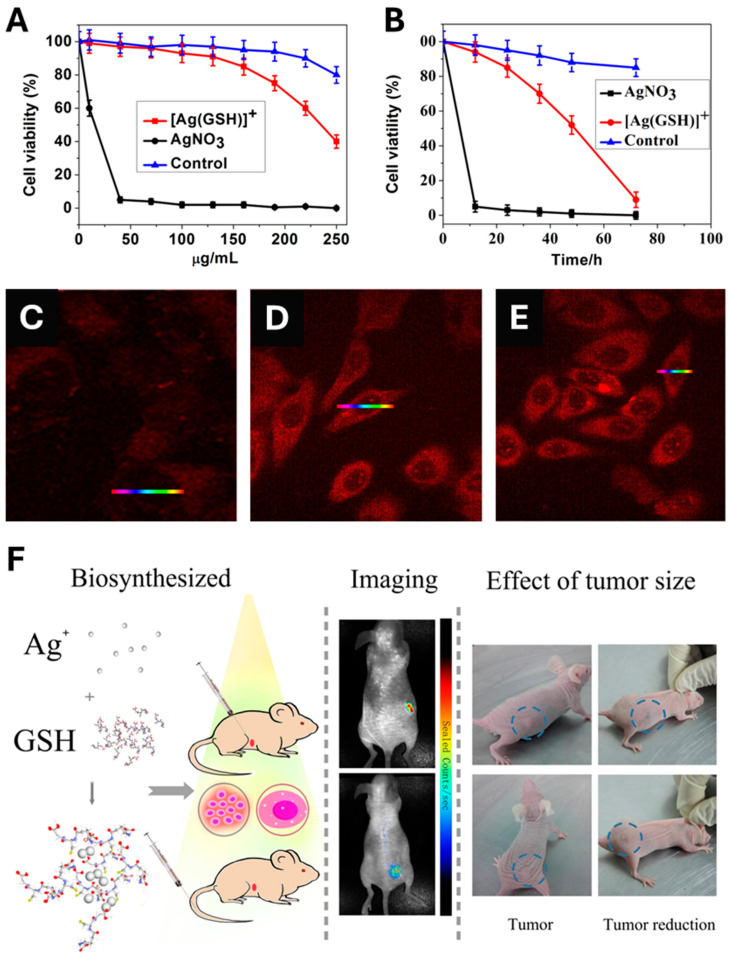
(**A**) Cytotoxicity of AgNO_3_ vs. [Ag(GSH)]^+^ (a silver derivative made of cationic silver and glutathione) at various concentrations towards HeLa cancer cells. The control is made of L02 normal cell line challenged by [Ag(GSH)]^+^. (**B**) Time-dependent toxicity of AgNO_3_ at 40 μg/mL and [Ag(GSH)]^+^ at 130 μg/mL towards HeLa cancer cells. The control is made of L02 normal cell line challenged by [Ag(GSH)]^+^ at 130 μg/mL. (**C**–**E**) Intracellular formation and accumulation of silver nanoclusters (AgNCs) within HeLa cancer cells as a function of administered concentration of [Ag(GSH)]^+^: (**C**) 0 μg/mL, (**D**) 40 μg/mL, and (**E**) 100 μg/mL. (**F**) A schematic depicting the in vivo formation of AgNCs in mice bearing tumor xenograft to which [Ag(GSH)]^+^ is injected either directly to the tumor or in the tail vein. AgNCs accumulate in the tumor which allows the tumor in vivo bioimaging owing to AgNC fluorescence and monitor the tumor size reduction owing to the action of the same AgNCs. Reproduced from Ref. [359] with permission from Springer Nature under the Creative Commons CC-BY-NC-SA license.

In contrast to the above-cited papers that typically, first, describe the biosynthesis of AgNPs; then, followed by their administration to finally monitor their in vivo anticancer activity, Gao et al. designed an original, single-step method for the in vivo synthesis of *bio*-AgNPs and their subsequent in vivo utilization [359]. Prior to the in vivo experiment, a comparative in vitro toxicity study of silver nitrate vs. a complex made by mixing silver nitrate and glutathione ([Ag(GSH)]^+^) was performed. As a result, [Ag(GSH)]^+^ shows no toxicity towards normal cells at all tested concentrations since the lowest cell viability exceeds 80% for the highest concentration tested (250 µg/mL) (Figure 7A). On the contrary, [Ag(GSH)]^+^ exerts a higher toxicity towards HeLa cancer cell line (Figure 7A). In addition, normal cells display a viability that exceeds 80% when challenged by 250 µg/mL of [Ag(GSH)]^+^ for 3 days while HeLa cells undergo a dramatic loss of viability (Figure 7B). Moreover, [Ag(GSH)]^+^ is found to penetrate cancer cells, thus offering the opportunity to implement near-infrared fluorescence imaging (NIR) whose intensity is dose-dependent (Figure 7C–E). Finally, the injection of [Ag(GSH)]^+^ into mice bearing xenograft tumors either via the tail vein or directly into the tumor yields the formation of silver nanoclusters (AgNCs), which exclusively occur within the tumor; this was confirmed by ex vivo analyses that found no presence of AgNCs in other parts of the body (Figure 7C–E). Owing to their NIR imaging features, these AgNCs allowed tumor imaging within a few hours after the injection of the complex (Figure 7F). Importantly, the tumors of mice treated with [Ag(GSH)]^+^ gradually receded until their total disappearance. Knowing their preferential accumulation in tumors, it is also possible to use *bio*-AgNPs, made using the ethanolic extract of *Zinnia elegans*, to implement NIR imaging and follow their biodistribution over time in tumors and vital organs, including the brain since the extract is known to accumulate in this organ [360].

In sum, *bio*-AgNPs are emerging as a potential anticancer therapeutic because of their preferential toxicity against tumors, in addition to their facile synthesis and low cost. Although most studies that report these significant anticancer effects of *bio*-AgNPs have been performed in vitro and much fewer in vivo animal models, there is hope that clinical therapeutic application of *bio*-AgNPs in the near future will position them as valuable agents in the fight against several human cancers [361], taking full advantage of the paradox of AgNP toxicity for cancerous and inertness for normal cells.

### 5.4. Exploitation of bio-AgNPs in Antibiofilm Action, Wound Healing and as Implant Coating

Antibiotics have gradually lost their activity due to their increasing and inappropriate use, leading to a major worldwide health concern as several strains of pathogenic bacteria do not respond to any available antibiotherapy following the evolutionary development of antibiotic resistance [362,363,364]. In addition to quorum sensing by which bacteria pass the information to their neighbors on circumventing the activity of antibiotics, microorganisms form biofilms in almost all environments which enable them to develop virulence factors and survive even in the presence of high concentrations of antibiotics [363,365,366]. Bacterial biofilms are structured clusters of cells or colonies embedded within a polymeric extracellular matrix and attached to a surface; this matrix acts as a barrier that hinders the action of host immune cells and the delivery of antibiotics should these bacteria be still active, therefore providing them protection by creating the suitable conditions for their survival, proliferation and spreading towards other parts of the body [367].

The presence of opportunistic pathogens in the form of variously structured biofilms is also a hallmark of most wounds, including chronic ones and others associated with burns or specific pathologies, such as diabetes. It is in these situations, research on the use of antimicrobial NPs, including AgNPs, has shown significant promise for combating infection and leading to wound healing. Choosing the right NP-based treatment depends on the wound’s specific requirements and biofilm type. Integrating NPs into wound dressings can improve therapeutic outcomes and reduce antibiotic use, thus addressing the serious challenge of antibiotic-tolerant infections. Ultimately, these strategies aim to replace or greatly lower antibiotic doses for managing biofilm infections [368].

Similarly, various commercially available accessories and devices improve the patients’ quality of life, such as prostheses and implants used to repair teeth, bones, and limbs, reestablish human mechanical functions, and improve mobility, comfort, and autonomy [369]. However, microbial infections and the subsequent biofilm formation on their surface constitute serious concerns as they potentially activate the patient’s immune response, induce implant rejection, and even cause septicemia in the most severe cases [370,371]. This can prove insurmountable with current antibiotics, necessitating the replacement of the implants/prostheses [372]. To prevent any biofouling, surface colonization and biofilm formation, the most widespread and relevant solution consists of the surface modification of implants and prostheses using coatings made of antibiotic-loaded polymers or inorganic NPs [372,373]. Among the latter, *bio*-AgNPs appear as a suitable and versatile solution thanks to the ease of their synthesis and casting on surfaces, and the large spectrum of their sustained biological activity against several microorganisms, including both gram+ and—bacteria, yeast, and fungi [294,374,375]. *bio*-AgNPs are also attracting interest to be incorporated into water filtering systems [376,377,378].

Given their well-documented and outstanding biocidal activity, several methodologies enable the exploitation of *bio*-AgNPs in the battle against the spread of pathogenic microorganisms and emerging health complications [294,379]. Achieving the same performance as their chemically synthesized analogs [380], *bio*-AgNPs could be directly cast on the surface of implants and prostheses since they prevent biofilm formation and inhibit microbial spreading [381,382,383,384]. They can also be incorporated into membranes, matrices, composites, and wound dressings [14,385]. Lastly, *bio*-AgNPs can also be topically applied to wounds and burns [386].

The antimicrobial activity of *bio*-AgNPs is size-dependent [351] and may stem from several factors, such as ROS generation, disruption of the cell membrane, protein denaturation and/or leakage, and cell lysis [159,387,388,389]. Spherical *bio*-AgNPs, prepared from *Miscanthus khasiana* plant extract and self-assembled within amine monolayers inhibit bacterial adhesion and biofilm formation of *P. aeruginosa* by reducing its viability by 67% [390]. *bio*-AgNPs prepared using the officinal plant *Tinospora cordifolia* reach 83% biofilm reduction in S. *aureus*, exhibiting a higher performance when compared to the antibiotic tetracycline or the extract used from the same plant [391]. Importantly, this effect lasts in time even after the removal of the NP treatment. Owing to ROS generation and impairment of cell membrane integrity, *bio*-AgNPs prepared using *Anabaena variabilis* cell extract disrupt *Candida albicans* biofilms in a dose-dependent manner [392]. In addition, a synergistic effect is observed by combining the same NPs with antibiotics [393]. Concretely, this drastically lowers the minimal inhibitory concentrations (MIC) of both the *bio*-AgNPs and antibiotics used to combat the tested bacterial and yeast strains, although its extent remains species- and strain-dependent. For instance, the MIC of *bio*-AgNPs and amphotericin B are, respectively, 12.5 and 3.12 µg/mL when used separately against *C. albicans*. It falls to 3.12 and 0.09 µg/mL, respectively, when combined. In the case of *P. aeruginosa*, the MIC plummets from 6.25 and 2.3 µg/mL of *bio*-AgNPs and streptomycin, respectively, when used separately, to 1.56 and 0.39 µg/mL of *bio*-AgNPs and streptomycin, respectively, when used in combination. Other studies confirm the observed synergistic effect [181,303,394,395,396]. A similar synergy is obtained by combining *bio*-AgNPs with peptides [397], metabolites [398] or a biosurfactant [399]. In all cited studies, the antimicrobial activity of *bio*-AgNPs is always and significantly much higher than that of the extract used [325,391,396,400]. Moreover, no clear trend is observed regarding the antibacterial effect of bio-AgNPs vs. antibiotics in general, since contradictory results have been reported [294,391,401]. However, a clear tendency advocates for *bio*-AgNPs in terms of antimicrobial efficacy when compared to their chemical/commercial counterparts [192,399,402].

**Figure 8:**
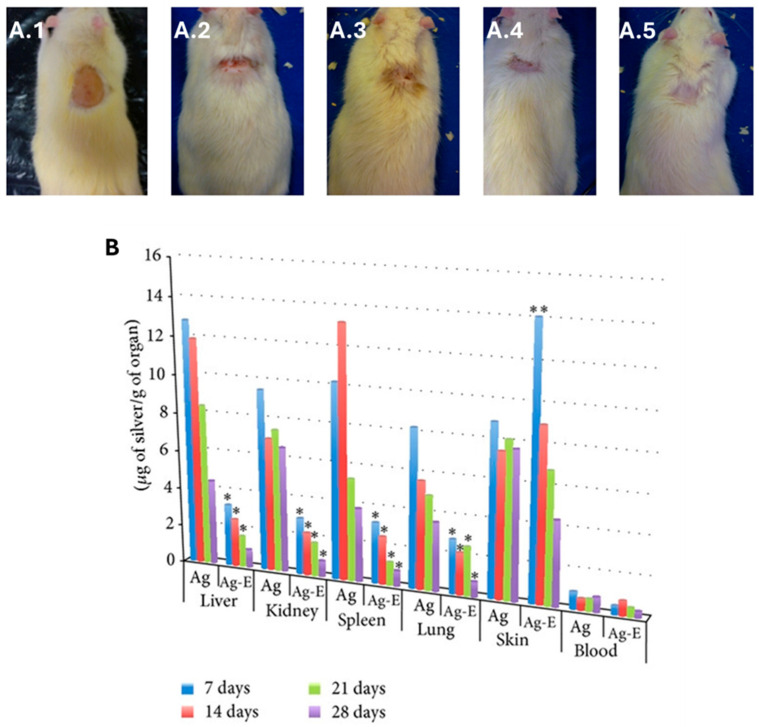
(**A**) Synergistic effect of *bio*-AgNPs, synthesized using the aqueous filtrate of *Fusarium oxysporum*, and Enoxaparin (Enox), an anticoagulant drug, on wound healing in male Wistar rats subjected to thermal injury: (**A.1**) provoked burn injury; (**A.2**) control rat, untreated after 28 days of burn injury; (**A.3**) *bio*-AgNP-treated rat after 28 days of burn injury; (**A.4**) Enox-treated rat after 28 days of burn injury; and (**A.5**) *bio*-AgNP + Enox-treated rat after 28 days of burn injury. (**B**) Silver concentration monitoring at different times in organs in rats treated with *bio*-AgNPs (Ag) and with *bio*-AgNP + Enox (Ag-E). Statistical significance is denoted as * *p* ≤ 0.001 for AgNP-Enox vs. AgNPs, ** *p* ≤ 0.01 for AgNP-Enox vs. AgNPs. Adapted from Ref. [411] with permission from Wiley under Creative Commons CC BY 3.0.

*bio*-AgNPs may be topically applied as ingredients of formulations for different types of wounds aiming to accelerate the in vivo healing process by combating microbial infections and reducing inflammation [403,404,405,406]. Importantly, this healing efficacy may equal that of the conventional medication, Betadine^TM^ [407]. The same healing effect is achieved when *bio*-AgNPs are part of hydrogels applied in rats bearing 6 mm diameter excision wounds [408]. A similar effect is obtained when *bio*-AgNPs enter the composition of an ointment used to treat induced burn wounds in rats by efficiently reducing the wound size, decreasing the epidermis layer, and lowering mast cell migration due to the proper regulation of inflammatory factors [167]. This healing effect of *bio*-AgNPs is corroborated in rats bearing not only an induced wound but also subjected to *P. aeruginosa* infection [405,409,410]. It is also possible to reach a synergistic effect by combining *bio*-AgNPs with the low-molecular-weight heparin enoxaparin (Enox) [411]. As displayed in Figure 8A, the best wound contraction was attained using *bio*-AgNPs + Enox (95%) when compared to the control (55%), *bio*-AgNPs alone (89%), and Enox alone (91%). In addition, the silver concentration in explored organs (liver, kidney, lung, and spleen) was lower in the group treated with *bio*-AgNPs + Enox than with *bio*-AgNPs (Figure 8B). An opposite trend was observed for skin until day 7 following the burn. On the other hand, silver concentration in blood was very low for both formulations, suggesting an elimination process that involves liver and spleen.

In addition to the previously mentioned antimicrobial effects of AgNPs via ROS generation and cell membrane disruption, the facilitation of Ag^+^ release by wound exudates may lead to the disruption of DNA replication and death of the microbial pathogens [412].

The effect of the specific AgNP formulation (e.g., in terms of polymeric matrix, colloidal status, etc.) on antibiofilm and wound-healing applications has been studied recently, indicating significant potential, as can be seen from the following examples. Chitosan-stabilized AgNPs tested against *S. aureus* and *P. aeruginosa* biofilms led to biofilm reduction by 48% and 78%, respectively [413]. Colloidal AgNPs tested in patients with infections by methicillin-sensitive and methicillin-resistant *S. aureus* (MSSA, MRSA), *Enterococcus faecalis*, and vancomycin-resistant Enterococci (VRE) were found to inhibit biofilm formation in the low, medium, and high biofilm producers by 91%, 83%, and 75%, respectively, when applied at the highest concentration of 52 ppm [414].

Casting or grafting *bio*-AgNPs onto surfaces constitutes an explored approach in combating biofilm formation and microbial colonization of surfaces, surgical sutures, catheters and wounds [415,416,417,418]. For instance, the efficacy of *bio*-AgNPs deposited on alumina disks in preventing colony formation is improved thanks to the coupling action of the extract used to produce them [381]. This resulted in a less wettable and very rough surface that reduced the number of colony formation units (CFUs) by 90–99.9% for the microbial isolates tested when compared to the bare alumina disks. On the other hand, the modified surface maintains a valuable biocompatibility towards human cell lines. These findings showcase the potential of *bio*-AgNPs in the design of antibiofouling yet biocompatible materials. Other findings show that coating a dental acrylic resin with *bio*-AgNPs yields a significant reduction in C. *albicans* biofilm formation [419]. It is also possible to modify cotton and polyester fabrics with *bio*-AgNPs to endow them with a large spectrum biocidal activity while maintaining, at the same time, a good biocompatibility towards moth larvae [420]. Interestingly, cotton fibers impregnated with *bio*-AgNPs maintain their biocidal activity against yeast and bacteria even after repeated wash cycles [421]. Other findings demonstrate that a firm anchoring of *bio*-AgNPs in a bandage gives better results regarding the wound healing activity [422]. In contrast to the above encouraging studies, there are also recent reports raising doubts about the presumed generality of AgNP antimicrobial and wound healing efficacy, depending on the context, with some works even suggesting the development of Ag resistance. For instance, an adverse effect of Ag in wound healing due to DNA damage of local skin and immune cells was recently reported [98]. The basis for potential tolerance of Ag by some pathogens goes back to the discovery in 2009 of Ag resistance genes in methicillin-resistant *S. aureus* (MRSA) and methicillin-resistant coagulase-negative staphylococci (MR-CNS) isolated from wound and nasal sources [423]. Therefore, there is a real need for further context-focused research into AgNP-based antibiofilm and wound healing technologies.

## 6. Environmental Applications of *bio*-AgNPs

Environmental pollution is one of the biggest global issues due to the damage it causes to ecosystems and human health. Amongst its numerous sources, there are industries that discharge substantial amounts of contaminants like heavy metals, oils and dyes [424]. Industrial effluents, carrying heavy metals, such as Pb^2+^, Zn^2+^, Cu^2+^, Hg^2+^ and Ni^2+^, directly and indirectly commingle with both groundwater and surface water sources, endangering flora and fauna [425,426]. Different methods, such as chemical precipitation, adsorption, ion exchange, membrane filtration and electrolytic methods, have been developed for the removal of these contaminants [426,427]. Despite their efficiency in removing contaminants, most of these methods are neither eco-friendly nor can they remove all types of contaminants, not to mention the emerging ones, which are often harmful at very low concentrations (micropollutants). Hence, the use of metallic NPs appears as an alternative solution, thanks to their properties like high affinity, specificity, and a large surface area. NPs possess a high affinity to many types of contaminants, especially in water and wastewater [427]. Various NPs including AgNPs are currently being used in water and wastewater treatment technologies not only for their efficacy but also for their sustainability and eco-friendliness, especially when they are produced using benign biological sources. As pointed out above, the major advantages of the biosynthesized AgNPs are their high surface area for binding and specific affinity for metallic ions [424]. As a consequence of their superior qualities, *bio*-AgNPs are often used in combination with other agents, such as organic materials, membranes, fibers, and alloys to widen the spectrum of their environmental applications [36]. For instance, studies on hybrid composites made of *bio*-AgNPs and cellulose show that when used as a coated filter paper with the correct concentration, they can attain up to 100% removal of *E. coli*, thus proving to be an easy-to-use material in emergency antibacterial water filters [428].

Nowadays, the main problem with conventional water purification or disinfection using chemicals consists of the generation of potentially ecotoxic disinfection by-products, commonly referred to as DBPs [429]. Given their antimicrobial properties, *bio*-AgNPs appear to be effective water disinfectants, hence avoiding the generation of the harmful DBPs [430]. One of the novel uses of *bio*-AgNPs in water treatment lies in membrane technology. *bio*-AgNPs can significantly modify the membrane properties, leading to enhanced performance. For example, Wu et al. produced *bio*-AgNPs by reducing and capping cationic silver ions using bacteria [431]. Then, they embedded the resulting *bio*-AgNPs within polysulfone substrate to prepare a thin-film composite toward osmosis membranes, which resulted in refined porosity, greater water flux, enhanced surface hydrophilicity, and better antibacterial and antifouling properties compared to their pristine counterpart. Moreover, silver nanocomposite-activated carbons are currently being researched for the treatment of emerging drinking water contaminants, such as per- and poly-fluoroalkyl substances (PFAS) [432]. Known as “forever chemicals” for their persistence in the environment, these chemicals are major human health concerns owing to their toxicity and bioaccumulation potential [433]. Although specific studies pertaining to the use of biogenic nanomaterials including AgNPs for treating PFAS are still unavailable, the need for eco-friendly biogenic treatment approaches is critical as more of these chemicals emerge into the environment. In addition to their use in treatment technologies, *bio*-AgNPs have been widely used in sensor systems to monitor water pollution [434,435]. The straightforward and versatile surface functionalization of *bio*-AgNPs enables their selective use for specific analytes within sensor systems. Various sizes of *bio*-AgNPs with various morphologies and surface functionalities have been studied for water pollution monitoring and treatments (Table 1).

In addition to drinking water treatment, *bio*-AgNPs have found their way into wastewater treatment [450]. For example, *bio*-AgNPs are effective in removing major industrial contaminants, including toxic chemicals from textile wastewater [446,447]. Other applications rely on the catalytic properties of the *bio*-AgNPs to speed up the rate of reduction of selective textile-industry dyes and for the efficient removal of toxic non-biodegradable organic dyes, respectively [438,447]. Moreover, *bio*-AgNPs produced using two classes of fungi (*Penicillium citreonigrum* and *Scopulariopsis brumptii*) are used as an antimicrobial agent for the low-cost removal of pathogens from wastewater [451]. Since *bio*-AgNPs are produced using various natural bioresources, this eliminates the use of chemicals as reducing or stabilizing agents.

The rapid and precise detection of highly toxic heavy metals, such as As, Cd, Pb, Hg, Cr, and their derivatives, is strongly enabled using *bio*-AgNP-based sensors [38]. Furthermore, the simplicity, accuracy, and low cost of *bio*-AgNP-based colorimetric/plasmonic nanosensors are major assets in the detection of many other environmental pollutants in water [452]. Although *bio*-AgNPs have seen their widest use in water and wastewater treatment, it is obvious that their use will continue to expand in other areas of environmental application. A recent study clearly demonstrated that *bio*-AgNPs reduce the stress in plants that arises from environmental pollution [453]. Therefore, *bio*-AgNPs can play a vital role by mitigating both the cause and the effect of the ongoing environmental pollution. In sum, it is worth observing that the surface functionalities of *bio*-AgNPs can lead towards a sustainable solution to water pollution.

## 7. Conclusions and Perspectives

As amply documented from this extensive critical survey, the status of AgNPs is becoming consolidated in a range of biological (including health-related) and environmental applications thanks to their distinctive characteristics, such as their tiny size and commensurate high surface-to-volume ratio, surface-modifying capacity, stability with low chemical reactivity, biocompatibility, and biosafety. These attributes, together with appropriate conditioning from green biological synthesis, are placing *bio*-AgNPs at center stage for biosensing, drug delivery, antimicrobial and cancer therapy, and pollution control, justifying the welcome paradox of these nano-objects’ specificity (e.g., towards pathogens or malignancies) together with their inertness for non-target organisms and tissues.

The remarkable properties of *bio*-AgNPs need to be explored further since, in combination with synergistic formulations, the range of their applications is constantly extended. Because of increasing human exposure, the issue of their biosafety is bound to be scrutinized more thoroughly, with suggestions for complete and systematic genotoxicological characterization of AgNPs [454]. A recent massive evaluation of hundreds of studies over the last 20 years on metal nanoparticles including AgNPs found that *bio*-AgNPs exhibit lower genotoxicity compared to those synthesized by conventional methodologies [455]. In this connection, the combination of AgNPs with selected conventional chemotherapeutics aiming at synergistic antiproliferative effects promises to offer, as a bonus, lower genotoxicity as shown recently by extensive nanotoxicological assessment of AgNPs combined with tamoxifen on breast tumor cells [456].

In the area of combatting wound infections through the beneficial action of *bio*-AgNPs, efforts will increase in several complementary directions, where AgNPs become components of integrated solutions that include composite nanomaterials, flexible nanoplatforms, and minimally invasive nanodevices. The intelligent targeting of infections caused by methicillin-resistant *S. aureus* (MRSA) with acidic biofilms and alkaline wound microenvironments has recently been achieved by devising an acid−base responsive bionic claw microneedle (MN) loaded with Au@ZnO/Ag (AZA) core−shell nanoparticles [457]. Wound healing was promoted through the synergy of released Zn^2+^/Ag^+^. The beneficial role of Ag^+^ is emerging as an essential part of “silver nanomix”, a highly cost-effective new-generation nanomaterial for wound dressing developed from the systematic hybridization of metallic AgNPs with ionic Ag [458]. On the other hand, the MN component of the wound-healing solution can be loaded with sophisticated nano-entities like silk fibroin microspheres (SFMs) in combination with AgNPs and antibiotics for simultaneous delivery into biofilms to facilitate synergistic therapeutic effects and eventually eradicate bacterial biofilm infections [459]. Non-antibiotic approaches to fight infection are generating novel multifunctional nanoplatforms, as illustrated by the combined introduction of pH-dependent and ROS-scavenging antibacterial, anti-inflammatory, and proangiogenic properties to promote wound healing by integrating allicin (Ac) into an AgNP-loaded zeolite imidazole framework collectively termed Ac@ZIF-8/AgNPs [460].

The path to clinical and industrial translation for *bio*-AgNPs is fraught with several severe technical and regulatory bottlenecks. An important issue is the reliable attainment of reproducibility and control of batch-to-batch variation. Since biogenic synthesis uses complex, often non-standardized biological resources like plants, microbes or their extracts, variations in the source material, pH, or temperature at scale may result in inconsistent particle size, shape, and surface chemistry, which, in turn, could affect the nanoparticles’ efficacy and safety. This variability is incompatible with the stringent rules mandated by regulatory bodies like the US Food and Drug Administration (FDA) and the European Medicines Agency (EMA), which require strict adherence to Good Manufacturing Practice (GMP) standards to ensure a consistent, well-characterized product. An additional issue is the cost of downstream processing towards purification of the clinical- or industrial-grade product. The various separation and purification steps to remove residual biological components and unreacted precursors from the crude biogenic nanoparticulate formulation may even diminish or eliminate the initial cost advantage over conventional synthesis routes. Addressing these challenges necessitates the establishment of standardized protocols, the implementation of advanced Process Analytical Technology (PAT) for real-time quality control, and the development of more economical, high-throughput purification methods compatible with clinical production or commercial biomanufacturing.

Finally, a new front in the quest for ever more efficient antimicrobial nanoparticles, including AgNPs, for bioapplications is the necessity to address concerns about the emergence of microbial resistance, which could compromise their effectiveness as innovative solutions replacing antibiotics. The development of targeted interventions addressing the putative evolutionary mechanisms of nanoresistance development in bacteria could lead to its practical elimination. In one recent example, the resensitization of bacteria against AgNPs was achieved via physicochemical strategies blocking energy supply and neutralizing bacterial intracellular acidic pH [461].

## Figures and Tables

**Table 1:** Removal of environmental pollutants using *bio*-AgNPs.

Natural Biomass Used	Size/nm	Removal Technique	Pollutants Removed *	Ref.
Aqueous extract of *Acacia nilotica* leaves	10–20	Catalytic degradation	Methylene blue and Congo red	[436]
Fruit extract of *Sterculia acuminata*	~10	Catalytic degradation	Organic dyes, such as 4-nitrophenol, methylene blue (100%), methyl orange, phenol red, and direct blue 24	[437]
Fruit extract of *Viburnum opulus*	7–26	Catalytic degradation	Tartrazine, carmoisine, and brilliant blue FCF	[438]
*Cuphea procumbens*	~24	Photocatalytic degradation	Congo red (~87%) and malachite green (~82%)	[439]
Extract of *Convolvulus arvensis* leaves	~28	Catalytic degradation	Methylene blue	[188]
Extract of *Diospyros lotus* leaves	~20	Catalytic degradation	Methylene blue	[440]
Aqueous extract of *Areca catechu*	18–24	Catalytic degradation	Organic pollutants, such as methylene blue, eosin-yellowish, methyl orange, and 4-nitrophenol	[441]
*Solanum tuberosum* infusion	10–12	Photocatalytic degradation	Methyl orange (up to ~70% within 8 h)	[442]
Extract of *Aegle marmelos* leaves	5–30	Photocatalytic degradation	Methylene blue (up to ~50% within 3 h)	[209]
Extract of *Euphorbia geniculata* leaves	~17	Catalytic degradation	Methyl orange (16–97%, depending on concentration)	[443]
*Mentha piperita*	~15	Sorption	Crystal violet (70–100%, depending on pH)	[444]
Fruit extract of *Berberis integerrima*	~30	Photocatalytic degradation	Methylene blue (up to 82.5% within 75 min)	[445]
*Bacillus marisflavi*	11–39	Photocatalytic degradation	Textile effluents including synthetic azo dyes, such as direct blue-1, methyl red, and reactive black-5	[446]
Peel extract of *Citrus paradisi*	~15	Catalytic degradation	Congo red, methylene blue (~93%), malachite green (~84%), rhodamine B (~61%) and 4-nitrophenol (~89%)	[447]
*Aloe vera* extract	3–14	Heavy metal removal	Hg(II) (>95%)	[448]
*Lactobacillus fermentum*	~11	Anti-biofouling	Inhibition of bacterial growth and attachment to the surface of polyethersulfone membranes	[449]
*L. fermentum*	~6	Anti-biofouling	Improvement of antifouling properties of forward osmosis polysulfone membrane	[431]

* Removal efficiency is shown between () whenever this data was available.
